# Intention judgments are not a reliable measure of intuitive preferences

**DOI:** 10.1073/pnas.2516523122

**Published:** 2025-09-15

**Authors:** Tadeg Quillien

**Affiliations:** ^a^Department of Psychology, University of Edinburgh, Edinburgh EH89JZ, United Kingdom

Gervais et al. argue that people have an intuitive preference for religious belief, on the basis of an ingenious experiment on how people attribute intention (1). Participants read a story in which an editor accepts to publish an article on religion. The editor knows that publishing the article will cause more people to believe in God. But he does not care about that at all. He wants to publish the article because it will help sell more newspapers. The article is published, and more people start believing in God. Participants did not think that the editor *intended* to make people believe in God. But in a different experimental condition, the article is expected to cause *fewer* people to believe in God, and its publication decreases the number of people who believe in God. In that condition, people were slightly more likely to think that the editor intended to make people believe that God does not exist.

Gervais et al. argue that this asymmetry in intention attribution shows that people think it is bad to make people not believe in God. Past research has shown that people are more likely to say that an agent caused an outcome intentionally when that outcome is bad (2). So, if people are more likely to attribute intention to the editor who caused a decrease in religious belief, they must think (maybe at an implicit level) that religious belief is good.

An issue with this inference is that the relationship between moral valence and intentionality judgments is complex. Attributing intentionality involves sophisticated causal and counterfactual computations (3, 4). Moral valence might shape intention attribution only indirectly, by influencing these more basic computations (2, 3). And many factors besides moral valence or preferences influence people’s judgments (5).

Consistent with this perspective, it is not always the case that people attribute more intentionality to bad outcomes. In one study, participants were told that the CEO of a corporation in Nazi Germany refused to implement a new policy that would make it easier to send people to concentration camps. People judged that the CEO intentionally refused to implement the policy. In a condition where the CEO complied with the policy, participants were less likely to judge that he did so intentionally (6).

In another study, a character prevents the explosion of a nuclear reactor by correctly guessing, in a feat of incredible luck, the 10-digit password of a computer. Participants judged that the agent intentionally prevented the nuclear explosion. But in a condition where correctly guessing a 10-digit code causes the agent to win a lottery, participants were unlikely to say that the agent intentionally won the lottery (7).

It would be strange to conclude from these data that people have an intuitive preference for sending people to concentration camps, or an intuitive preference for nuclear explosions. In sum, while the cross-culturally robust asymmetry in intention attribution uncovered by Gervais et al. is interesting, it might be premature to draw strong conclusions from that finding.
